# The Association of a SNP Upstream of *INSIG2* with Body Mass Index is Reproduced in Several but Not All Cohorts

**DOI:** 10.1371/journal.pgen.0030061

**Published:** 2007-04-27

**Authors:** Helen N Lyon, Valur Emilsson, Anke Hinney, Iris M Heid, Jessica Lasky-Su, Xiaofeng Zhu, Gudmar Thorleifsson, Steinunn Gunnarsdottir, G. Bragi Walters, Unnur Thorsteinsdottir, Augustine Kong, Jeffrey Gulcher, Thuy Trang Nguyen, André Scherag, Arne Pfeufer, Thomas Meitinger, Günter Brönner, Winfried Rief, Manuel E Soto-Quiros, Lydiana Avila, Barbara Klanderman, Benjamin A Raby, Edwin K Silverman, Scott T Weiss, Nan Laird, Xiao Ding, Leif Groop, Tiinamaija Tuomi, Bo Isomaa, Kristina Bengtsson, Johannah L Butler, Richard S Cooper, Caroline S Fox, Christopher J O'Donnell, Caren Vollmert, Juan C Celedón, H. Erich Wichmann, Johannes Hebebrand, Kari Stefansson, Christoph Lange, Joel N Hirschhorn

**Affiliations:** 1 Program in Genomics, Divisions of Genetics and Endocrinology, Children's Hospital, Boston, Massachusetts; 2 Program in Medical and Population Genetics, Broad Institute of Harvard and MIT, Cambridge, Massachusetts; 3 Department of Pediatrics, Harvard Medical School, Boston, Massachusetts; 4 deCODE genetics, Reykjavik, Iceland; 5 Department of Child and Adolescent Psychiatry, Rheinische Kliniken Essen, University of Duisburg-Essen, Essen, Germany; 6 Institute of Epidemiology, GSF-National Research Centre for Environment and Health, Neuherberg, Germany; 7 Institute of Medical Informatics, Biometry, and Epidemiology, Ludwig-Maximilians-Universität München, Munich, Germany; 8 Channing Laboratory, Brigham and Women's Hospital, Harvard Medical School, Boston, Massachusetts, United States of America; 9 State University of New York Upstate Medical University, Syracuse, New York, United States of America; 10 Department of Epidemiology and Biostatistics, Case Western Reserve University, Cleveland, Ohio, United States of America; 11 Institute of Medical Biometry and Epidemiology, Philipps-University of Marburg, Marburg, Germany; 12 Department of Clinical Psychology and Psychotherapy, Philipps-University of Marburg, Marburg, Germany; 13 Institute of Human Genetics, Technical University Munich, Munich, Germany; 14 Institute of Human Genetics, GSF-National Research Center for Environment and Health, Genome Analysis Center, Neuherberg, Germany; 15 Division of Pediatric Pulmonology, Hospital Nacional de Niños, San José, Costa Rica; 16 Department of Endocrinology, University Hospital MAS, Lund University, Malmö, Sweden; 17 Department of Medicine, Helsinki University Central Hospital, University of Helsinki, Helsinki, Finland; 18 Research Program for Molecular Medicine, University of Helsinki, Helsinki, Finland; 19 Folkhalsan Research Center, Helsinki, Finland; 20 Department of Preventive Medicine and Epidemiology, Loyola University Medical Center, Maywood, Illinois, United States of America; 21 Framingham Heart Study, National Heart, Lung and Blood Institute, Framingham, Massachusetts, United States of America; 22 Department of Genetics, Harvard Medical School, Boston, Massachusetts, United States of America; University of Michigan, United States of America

## Abstract

A SNP upstream of the *INSIG2* gene, rs7566605, was recently found to be associated with obesity as measured by body mass index (BMI) by Herbert and colleagues. The association between increased BMI and homozygosity for the minor allele was first observed in data from a genome-wide association scan of 86,604 SNPs in 923 related individuals from the Framingham Heart Study offspring cohort. The association was reproduced in four additional cohorts, but was not seen in a fifth cohort. To further assess the general reproducibility of this association, we genotyped rs7566605 in nine large cohorts from eight populations across multiple ethnicities (total *n* = 16,969). We tested this variant for association with BMI in each sample under a recessive model using family-based, population-based, and case-control designs. We observed a significant (*p* < 0.05) association in five cohorts but saw no association in three other cohorts. There was variability in the strength of association evidence across examination cycles in longitudinal data from unrelated individuals in the Framingham Heart Study Offspring cohort. A combined analysis revealed significant independent validation of this association in both unrelated (*p* = 0.046) and family-based (*p* = 0.004) samples. The estimated risk conferred by this allele is small, and could easily be masked by small sample size, population stratification, or other confounders. These validation studies suggest that the original association is less likely to be spurious, but the failure to observe an association in every data set suggests that the effect of SNP rs7566605 on BMI may be heterogeneous across population samples.

## Author Summary

Obesity is an epidemic in the United States of America and developing world, portending an epidemic of related diseases such as diabetes and heart disease. While diet and lifestyle contribute to obesity, half of the population variation in body mass index, a common measure of obesity, is determined by inherited factors. Many studies have reported that common sequence variants in genes are associated with an increased risk for obesity, yet most of these are not reproducible in other study cohorts, suggesting that some are false. Recently, Herbert et al. reported a slightly increased risk of obesity for people carrying two copies of the minor allele at a common variant near *INSIG2*. We present our attempts to further evaluate this potential association with obesity in additional populations. We find evidence of increased risk of obesity for people carrying two copies of the minor allele in five out of nine cohorts tested, using both family- and population-based testing. We indicate possible reasons for the varied results, with the hope of encouraging a combined analysis across study cohorts to more precisely define the effect of this *INSIG2* gene variant.

## Introduction

Body mass index (BMI) is a heritable measure of obesity that is routinely obtained in large cohorts, is correlated with other measures of obesity, and predicts morbidity and mortality from obesity-related diseases [1–4]. Thus, BMI is a readily accessible trait that can be used to screen for genetic variants that increase an individual's risk for obesity and its complications. There have been more than one hundred publications reporting association between common genetic variants and BMI, but few of the associations have been reproducible in multiple populations [5]. Genotyping of variants has increased exponentially in scale over the past few years, and much more comprehensive screens of common genetic variation for association with obesity are now possible. The poor rate of reproducible findings in association studies in general and obesity in particular are likely due to a combination of false-positive results, underpowered attempts to reproduce associations with modest effects, systematic bias due to technical artifacts or population stratification, and perhaps true heterogeneity in effect across populations due to differences in genetic or environmental modifiers [6,7]. Thus, new reports of association require rapid, well-powered studies to validate true associations or identify false positives that could otherwise trigger unwarranted investigation of spurious findings.

Recently, Herbert and colleagues, including several of the authors of this study, reported a novel association between homozygosity for the minor allele of a single nucleotide polymorphism (SNP), rs7566605, and increased BMI [8]. The SNP has no known function, and the closest gene codes for the insulin signaling protein type 2 (INSIG2), a hijacking protein in the endoplasmic reticulum that, in response to changes in lipid levels, impedes the movement of sterol regulatory element binding proteins to the Golgi apparatus for processing and ultimately its release to act as a nuclear transcription factor and regulator of lipid biosynthesis [9–11]. Animal data suggests a role for INSIG2 in increasing triglyceride level in rats [12], as well as linkage to obesity phenotypes in mice [13].

The association of SNP rs7566605 with obesity was initially found in a set of related individuals from the Framingham Heart Study (FHS) offspring cohort [8]. The SNP was genotyped in five additional cohorts, and the association was observed again in four of these, including population-based studies, case-control samples, and family-based cohorts. However, no significant association was found in a fifth cohort (the Nurses Health Study [NHS]), where a slight trend in the opposite direction was seen. Approximately 10% of individuals were homozygous for the minor allele (C/C), and in a meta-analysis of the case-control samples (including the NHS cohort and excluding the FHS discovery cohort), these individuals had a 22% increased risk of obesity (defined as BMI ≥ 30 kg/m^2^). In the NHS cohort alone, the 95% confidence interval (CI) for the odds ratio (OR) for obesity was 0.58–1.13. Subsequently, two further groups reported no evidence of association in large cohorts, and a third found association only for people on the overweight end of their population [14–17].

We considered several possible explanations for observing an association in four cohorts but not in the fifth. The failure to observe association in the NHS sample could be due to more modest effects in this cohort and therefore inadequate sample size, population stratification, ascertainment bias, other unmeasured confounders, or any combination of these. It is also possible that evidence in the four cohorts was falsely positive, for any of a combination of reasons that could include hidden population substructure, technical artifacts, or statistical fluctuations causing false positives. However, because of the consistency across multiple cohorts, including studies with family-based design, we felt that these explanations were less likely. Finally, it is also possible that the association is heterogeneous across populations, either due to differences in ascertainment, or differences in genetic or environmental modifiers. Of these possibilities, it is most critical to assess first whether the original associations were spurious, so as to avoid further efforts expended on a false finding. Our primary objective was therefore to test additional large populations to evaluate further the validity and generalizability of this association. By studying these additional populations, including a sample with longitudinal data, we hoped to better assess the strength and consistency of the association between increased BMI and the risk genotype at rs7566605, and perhaps generate hypotheses about any inconsistencies in this association.

## Results

Descriptions of the cohorts used in this study are presented in Table 1, Table S1, and in the Methods. These nine cohorts are drawn from eight different populations and include a total of almost 17,000 individuals. The cohorts were not ascertained for BMI, except for the Essen study cohort, which was selected from the upper (BMI ≥ 30 kg/m^2^) and lower (BMI < 20 kg/m^2^) ends of the BMI distribution of their population and a portion of the African-American sample that was enriched for obese individuals. We tested for association with obese (BMI ≥ 30 kg/m^2^) versus non-obese (BMI <30 kg/m^2^) and also with BMI as a continuous trait, to mimic the association tests performed in the initial publication. All analyses were performed under a recessive model, with the prior hypothesis that C/C homozygotes would have a higher BMI than individuals in the other two genotype classes.

**Table 1:**
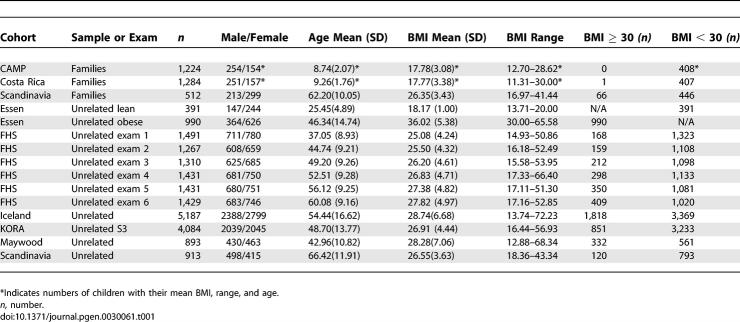
Eight Populations (*n* = 16,969) Used in Association Testing of rs7566605 and Obesity/BMI

The frequency of C/C homozygotes was increased in obese individuals compared to non-obese control individuals in several cohorts (Table 2). Nominally significant (two-tailed *p* < 0.05) associations between obesity (BMI ≥ 30 kg/m^2^) and the C/C were present in three samples: the Iceland cohort (OR = 1.29, 95% CI = 1.06–1.57, *p* = 0.0064), the Essen cohort (OR = 1.75, 95% CI = 1.15–2.68, *p* = 0.008), and in one of six exam cycles within the longitudinal data from the FHS cohorts (Table 2). In the Iceland cohort, the homozygote C/C genotype was associated with a 0.69 kg/m^2^ increment in BMI, which is in good agreement with the effect observed by Herbert et al. [1].

**Table 2:**
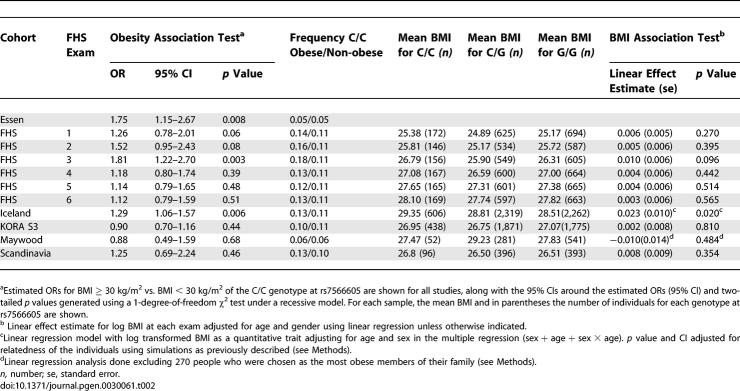
Association Studies of rs7566605 C/C Genotype and Obesity (BMI ≥ 30) and BMI as a Continuous Trait in Each of the Individual Unrelated Samples

The KORA S3, Maywood, and Scandinavian cohorts, and five of six exam cycles in the FHS cohort, did not show nominally significant associations under a recessive model. The Scandinavian, FHS, and Maywood samples may have been too small to achieve statistical significance with an association of the magnitude estimated by Herbert et al. (OR = 1.22). The Scandinavian cohort had an estimated OR (1.25) similar to the original report, but a *p* value of 0.46 and a wide 95% CI around the estimated OR (0.69–2.24). In particular, this cohort had only 120 people with BMI > 30 kg/m^2^, and the power to achieve nominal significance for an OR of 1.22 (as estimated in the original report) is only 15%. The estimated OR in the Maywood cohort was 0.88 but the CIs were also wide (*p* = 0.68, 95% CI = 0.49–1.59), which suggests that the sample was also underpowered to find this modest association and/or that the effect in this sample is smaller than in the original report.

The KORA S3 sample was much larger (851 obese and 3,233 non-obese), but had an OR of 0.90, with a 95% CI of 0.71–1.16, suggesting that the association is either more modest or absent in this cohort, limited to a particular subgroup of this population (see Discussion), and/or that when several samples are tested, some statistical fluctuation either away from or toward the null is expected. Association tests in the FHS cohort between the C/C genotype and obesity showed some apparent variability, achieving significance in some but not all of the six exams, with *p* values ranging from 0.003–0.51(Table 2); correcting the best *p* value for having tested six exams suggests that the totality of these findings are consistent with a replication (corrected *p* value = 0.018). There was no formal evidence of heterogeneity across the six exams (*p* = 0.47), and the 95% CIs for all exams include an OR of 1.22 (Table 2).

We also analyzed the five population-based samples—Maywood, Iceland, KORA S3, Scandinavia, and FHS (see Methods for details)—for association with BMI as a continuous trait, again under a recessive model controlling for age and gender. We saw similar results to those observed for the dichotomous analysis, with nominally significant associations between C/C homozygotes and increased BMI observed in the Iceland and FHS cohorts but not in KORA S3, Maywood, or Scandinavia (Table 2). When we analyzed association with BMI at each exam cycle from FHS separately, there was no significant evidence of association in a recessive model. The effect estimates trended in the same direction (exam 3, two-tailed *p* value = 0.096) (Table 2) as did estimates in the analysis using *z*-scores for BMI (see Methods) and mean *z*-score over six exams (unpublished data).

Finally, we tested SNP rs7566605 for association with increased BMI in three family-based samples, using PBAT [18]. Two of the three cohorts showed an association between SNP rs7566605 and BMI as a continuous trait under a recessive model (Table 3). (A dichotomous analysis was not done in these cohorts, because the definition of obesity we used for the remainder of the samples [BMI > 30 kg/m^2^] was not applicable to the children that made up a substantial part of each cohort.) The family-based portion of the Scandinavian cohort was composed of adults, but the incidence of obesity was only 13% (*n* = 66), limiting the power of a dichotomous analysis. Because BMI changes rapidly during childhood, we compared the results for the pediatric cohorts using three different measured outcomes: BMI, BMI adjusted for age and gender, and BMI-for-age percentile (Centers for Disease Control and Prevention 2000 National Center of Health Statistics); the *p* values for the corresponding FBAT statistics were essentially identical in each cohort (unpublished data).

**Table 3:**
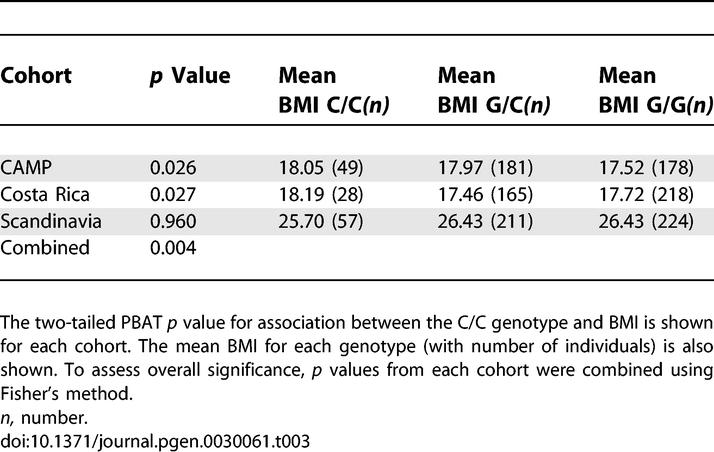
Association Studies of rs7566605 C/C Genotype Body Mass Index in Family Cohorts

To estimate the overall significance and effect size in the samples we studied, we performed a pooled analysis for both the unrelated and family-based cohorts. These combined analyses, which included both cohorts that showed association and those that did not, yielded independent, statistically significant associations for both the unrelated samples (Table 4) and the family-based samples (Table 3). Combining the *p* values of the family-based studies using Fisher's method provided evidence of replication (Fisher's combined *p* = 0.004; Table 3). For the unrelated samples (Table 4), we compared obese and non-obese people, and performed a combined analysis using each exam cycle of the FHS cohort in turn. Since the Essen cohort was ascertained as a severe obesity cohort with non-age matched controls, we tested for heterogeneity between studies using a modified Breslow-Day test [19,20]. There was evidence for heterogeneity when including the Essen cohort (*p* values for homogeneity = 0.007–0.08) so this cohort was excluded from the combined analyses. Mantel-Haenszel two-tailed *p* values ranged from 0.011 using FHS exam 3 to 0.054 using FHS exam 6 (Table 4). In these combined analyses, the estimated OR for obesity (BMI > 30 kg/m^2^) associated with the C/C homozygous genotype ranged from 1.13 to 1.18, somewhat lower than the effect size estimated by the original report [8]. There was also modest evidence of heterogeneity; *p* values for homogeneity ranged from 0.03 to 0.20, depending on which exam from FHS was included in the combined analysis (Table 4), suggesting that there might be some real variability in effect size across the samples in this study.

**Table 4:**
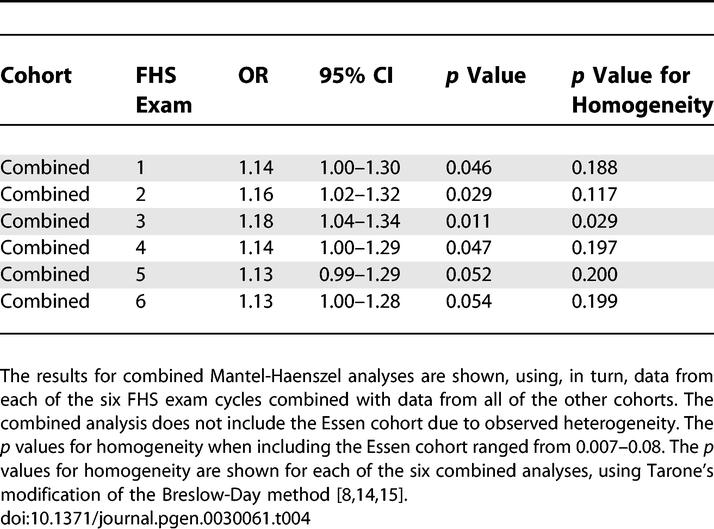
Association Studies of rs7566605 C/C Genotype in a Combined Analysis of All Urelated Samples Using One of Each FHS Exam Cycles

## Discussion

Association testing in these nine cohorts shows further evidence that individuals homozygous for the C/C genotype at SNP rs7566605 have a higher BMI and a higher risk of obesity. The association is detectable in diverse cohorts, in children as well as in adults, and in both family-based and population-based samples. The association is not likely to be due to stratification because it was seen in family-based samples such as Costa Rica and CAMP, which are immune to stratification, and because the original publication also described associations in family-based testing [8].

The effect of ascertainment on these analyses could potentially provide confounding of the association in four of these studies. Because index children in family-based studies in CAMP and Costa Rica were ascertained on the basis of asthma, a spurious association between SNP rs7566605 and BMI could be found if the SNP of interest was directly associated with asthma. However, none of the other cohorts were ascertained in this manner, lessening concerns about this source of bias as a potential cause of false-positive associations. In addition, the Scandinavian sample was ascertained as control subjects for a diabetes case control study (similar to the NHS in the original report). A further bias could potentially have been introduced by the selection of non-obese people in the Essen cohort who have a younger mean age than the obese people from this cohort (Table 1). The lean controls (mean BMI = 18.2 kg/m^2^) are less likely to be obese later in life, but a small portion of them could be misclassified as non-obese, which would tend to bias the estimate toward the null. Of note, the combined analysis remains significant even if we include this study (unpublished data).

The longitudinal nature of the FHS data may provide a clue to a possible cause for inconsistency in the association between SNP rs7566605 and obesity. In this cohort, a stronger effect on BMI was seen in the data from the first three exams than in the last three exams. The individuals at each exam are largely overlapping, making confounders less likely to explain a positive association in the early exam data and a lack of association in later data. Assuming that the association in this cohort is not a false positive due to statistical fluctuation, then the passage of time is the most likely explanation for the diminution of the association in this cohort. The decreasing evidence of association in theory could be due to an interaction with age, namely decreasing effect size with increasing age. Alternatively, a change in the environment could have diminished the strength of the association over time; this would be, in theory, consistent with a well documented “secular trend” of increased obesity over the relevant time period [21,22]. A preliminary and *post hoc* examination of the FHS data suggests that age may play an important role in modifying the strength of the association (unpublished data). This hypothesis would also be consistent with stronger effects in controls matched for early-onset disease (such as asthma) than in controls matched for later-onset diseases (such as diabetes). Finally, an additional *post hoc* analysis of the KORA S3 data suggests a stronger association in the most severely obese individuals (OR for BMI ≥ 38 kg/m^2^ was 1.78, 95% CI, = 0.99–3.21, *p* = 0.054), who perhaps became obese at an earlier age. Although these hypotheses are speculative at this time, they and other possibilities could and should be tested by a formal meta-analysis of our data, recent studies showing no association [14–16], and additional data that are likely to emerge. We (I.M.H. and colleagues) are in the process of organizing a meta-analysis to reexamine the *INSIG2* association in light of these hypotheses to better understand the relationship of this gene to obesity in the population.

In summary, the association of SNP rs7566605 with higher BMI is found in diverse populations. The number of studies in which a nominal association has been observed (five out of the nine cohorts reported here) appears more frequently than expected by chance. However, a more precise assessment of this apparent excess of associations will depend on the availability of a complete set of studies of this polymorphism. Large sample sizes were required to observe the association, but even some large samples have not demonstrated an association with this allele, possibly due to modification by age or other issues related to ascertainment. A combined analysis of both positive and negative studies presented here suggests that the association is valid but also suggests the possibility of heterogeneity across populations. Additional data, both positive and negative, ideally from large samples with good information regarding potential confounders and in a format suitable for meta-analysis, would be required to confirm the existence of heterogeneity and to further refine the estimate of the effect of this SNP on BMI in different populations. However, the evidence to date suggests that this variant has a detectable influence on BMI in a diverse range of populations.

## Materials and Methods

### Iceland cohort.

DNA samples were obtained from a large group of 5,187 Icelanders. The study group was composed of individuals who participated in studies of the genetic etiology of cardiovascular and metabolic diseases and the majority of these subjects were recruited as unaffected relatives of probands or as controls and did not have any history of metabolic or cardiovascular diseases. All participants in the study signed informed consent. All personal identifiers associated with tissue samples, clinical information, and genealogy were encrypted by the Icelandic Data Protection Authority, using a third-party encryption system in which the Data Protection Authority maintains the code [23]. Association testing was done according to that of the KORA S4 study design described in Herbert et al [1]. OR of genotype G1 (C/C) compared to genotype G0 (G/C + G/G) was calculated by [*n*(G1)/*m*(G1)]/[*n*(G0)/*m*(G0)], where *n* and *m* denote genotype counts in obese and non-obese individuals, respectively. The genotyping procedure has been previously described [24]. Genotype call rate was 97.3%. *p* value and CI were adjusted for relatedness of the individuals using simulations as previously described [25]. In each simulation, genotypes for the SNP are simulated through the Icelandic genealogy and the association test repeated treating those genotypes as real genotypes. By repeating this procedure 50,000 times we get the standard deviation of log(OR) under the null hypothesis of no association, which is used to calculate both the *p* value and the CI. We regressed the log transformed values for BMI on C/C carrier status by adjusting for age and sex in the multiple regressions as shown in Table 2.

### KORA S3 cohort.

In the Southern German region of Augsburg, which includes the city of Augsburg and the two surrounding counties, population-based surveys of the 25–74-y-old population were implemented in 1984 as part of the World Health Organization's Multinational Monitoring of Trends and Determinants in Cardiovascular Disease [MONICA]) project and continued since 1996 within the German Kooperative Gesundheitsforschung in the Region Augsburg (KORA) platform. The third survey, KORA S3, which was the study used in our analysis, was conducted in 1994–1995. Subjects (4,856) were recruited via registry according to the same protocol as the fourth survey (KORA S4) performed in 1999–2001, which was part of the initial replication samples in Herbert et al. The KORA surveys were described previously [22,26]. Genotyping was performed using a MALDI-TOF mass spectometry system (MassEXTEND; Sequenom, http://www.sequenom.com) and the call rate was 99.3%.

### FHS cohort.

DNA samples were obtained from 1,515 unrelated people from the offspring generation of the FHS [27]. We considered the possibility of overlap between the “unrelated plate” of the offspring cohort used here and with the family-based panel, approximately half of which was used in the analysis in the Herbert et al. report. There were 283 people who overlap between the “unrelated plate” and the full family-based panel, so these 283 people were excluded from the analyses reported here. The samples were genotyped using allele-specific primer extension of amplified products with detection by MALDI-TOF mass spectroscopy using a Sequenom platform as previously described [28–30]. Genotype call rate was 99.1% with no discordancies among replicate samples. Association testing was done with linear regression using BMI log transformed and adjusted for age and gender at all six exams.

### Maywood cohort.

DNA samples were obtained from 874 unrelated people, self-described as African-Americans, from the same cohort as was described in the original association report [8]. Unrelated people were selected from this population for genotyping. In 270 families, the most obese sibling was chosen to enrich the sample for obese people in the case-control comparison. These were not included in the quantitative trait analysis as described below in Statistical Analysis. Samples were genotyped as previously described [8,29]. Genotype call rate was 97.9% with no discordancies among replicate samples. Association testing was done with linear regression modeling of using log BMI corrected for age and gender with genotype in a recessive and additive model.

### Essen cohort.

DNA samples were obtained from 1,381 adults from Marburg, of which 990 were obese cases (BMI ≥ 30 kg/m^2^; mean BMI 36.02 ± 5.38 kg/m^2^) and 391 were lean controls (BMI ≤ 20 kg/m^2^, mean BMI 18.17 ± 1.00 kg/m^2^ [31]. Genotyping was carried out by PCR-RFLP with Bsp143I (digests the C-allele) (primers: 5′-TGAAGTTGATCTAATGTTCTCTCTCC-3′ and 5′-AAACCAAGGGAATCGAGAGC-3′). Association analysis under the recessive model, by χ^2^ testing.

### Costa-Rica cohort.

Nuclear families (415) of children with asthma in the Central Valley of Costa-Rica, a relative genetic isolate of predominantly Spanish and Amerindian ancestry [32,33]. Children and their families were enrolled as described previously [34] and anthropometric measurements of all probands included weight and height. However, this population was not ascertained based on morphometric phenotypes. Genotyping was performed using the Illumina BeadStation 500G system (http://www.illumina.com). Genotyping completion rate was >99.8% with no discordances among replicate genotypes. Of the 415 families with genotypic data, 408 had complete phenotypic data and were included in the analysis.

### Childhood Asthma Management Program.

The Childhood Asthma Management Program (CAMP) is a multicentered North American clinical trial designed to investigate the long-term effects of inhaled antiinflammatory medications in children with mild to moderate asthma [35]. Children ages 5 through 12 were eligible for inclusion in the study if they had a diagnosis of asthma and no other clinically significant conditions. Height and weight measurements were collected on these children during the prerandomization period. Of the 1,041 children originally enrolled, 968 children and 1,518 parents contributed DNA samples for genetic studies. Complete nuclear families (408) of self-described non-Hispanic white race with baseline BMI measurements are included here. Genotyping was performed using the Sequenom genotyping platform.

### Scandinavia cohorts.

The unrelated sample consisted of individuals from the Botnia Study chosen as control subjects from two cohorts to study diabetes. The first group were controls from a Scandinavian sample of 471 case-control pairs individually matched for gender, age, BMI, and geographic region in Sweden and Finland. The second group were from a Swedish sample of 514 case-control pairs who were individually matched for gender, age and BMI. Subjects were characterized as unaffected for diabetes by glucose tolerance testing as previously described [29]. The family cohort was comprised of 512 unaffected siblings from a Scandinavian sample of 1,189 siblings with and without diabetes, as previously described [36,37]. The samples were genotyped using by an allele-specific primer extension of amplified products with detection by MALDI-TOF mass spectroscopy using a Sequenom platform as previously described [28,29]. Genotype call rate was 96.5% with one Mendel error in one family and no discordancies among replicate samples.

### Statistical analyses.

The genotype data in each population was tested for deviation from Hardy-Weinberg and found to be consistent (*p* value > 0.01). Tests for association of rs7566605 with obesity were performed for the five population-based cohorts under a recessive model, classifying non-obese people as BMI < 30 kg/m^2^ and obese as BMI ≥ 30 kg/m^2^. Significance was assessed using a χ^2^ test with one degree of freedom and two-tailed *p* values were reported. The Mantel-Haenszel method was used for the combined analysis, and testing for heterogeneity was performed using the Breslow-Day test, as described previously [7,19,20].

For the four samples that had population-based components, an association analysis was performed using BMI as a continuous trait, adjusting for age and gender. A second analysis of the FHS cohort was done to make use of longitudinal data collected across six exams, approximately 4 y apart spanning 26 years from 1971–1997. For each exam, *Z* scores were calculated by the following process: within each decade of life and gender, log BMI was regressed against age. A *Z* score was then calculated for these age-adjusted BMIs based on the mean and standard deviation within each decade and gender for each exam. These were then analyzed using standard regression methods (implemented in SAS) for each exam individually, and also for the mean of all available *Z* scores across the six exams. For the KORA S3, Maywood, and Scandinavian cohort analyses we used standard linear regression with log transformed BMI and adjusted for age and gender. The linear regression analysis in the Maywood cohort excluded 270 people, who had been selected as the most obese person in their family, to avoid possible bias. The Iceland analysis was done with log transformed BMI as a continuous trait under a recessive model, adjusting for age and sex in the multiple regression (sex + age + sex × age).

Association testing of rs7566605 in the family-based cohorts was performed using the FBAT-approach as implemented in PBAT [18,38], with BMI as a quantitative (continuous) trait adjusted for age and gender by *Z* score under a recessive model. For the Costa Rica and CAMP populations, tests were also done for the outcome BMI adjusted for age and gender, and BMI-for-age percentile (Centers for Disease Control and Prevention 2000 National Center of Health Statistics). Because these studies were similarly sized, a combined analysis was performed using Fisher's method for combining *p* values, in which twice the negative sum of the natural log of *k* one-tailed *p* values is distributed as a χ^2^ distribution with 2*k* degrees of freedom [39]. In this method, a one-tailed *p* value for an effect in the opposite direction is first corrected by subtracting the *p* value from one; as all the effects in our studies were in the same direction, this correction was not necessary.

## Supporting Information

Table S1 Six Populations Divided into Non-obese (BMI<30 kg/m^2^) and Obese (BMI≥30 kg/m^2^) with Mean Age in years. (66 KB DOC)

### Accession Numbers

The National Center for Biotechnology Information (NCBI) (http://www.ncbi.nlm.nih.gov) accession numbers for the gene and gene product discussed in this paper are *INSIG2* (NM_016133) and INSIG2 (NP_057217).

## Abbreviations

BMI: body mass index
CI: confidence interval
FHS: Framingham Heart Study
NHS: Nurses Health Study
OR: odds ratio
SNP: single nucleotide polymorphism
