# Supplementary material for: The Association of a SNP Upstream of INSIG2 with Body Mass Index is Reproduced in Several but Not All Cohorts
Source: PLoS Genet. 2007 Apr 27;3(4):e61. doi: 10.1371/journal.pgen.0030061 (PMC1857727; doi:10.1371/journal.pgen.0030061)
Supplement: Table S1 — (66 KB DOC) [file pgen.0030061.st001.doc]

Table S1: Six populations divided into nonobese (BMI<30 kg/m^2^) and obese (BMI>30 kg/m^2^) with mean age in

| **Cohort** | **Sample**  **or Exam** | **N** | **Male/Female** | **Age Mean (SD)** | **BMI Mean (SD)** | **BMI range** |
| --- | --- | --- | --- | --- | --- | --- |
| Essen | Nonobese | 391 | 147/244 | 25.45(4.89) | 18.17 (1.00) | 13.71-20.00 |
| Essen | Obese | 990 | 364/626 | 46.34(14.74) | 36.02 (5.38) | 30.00-65.58 |
| FHS Exam 1 | Nonobese | 1323 | 610/713 | 36.89 (8.97) | 24.04 (3.00) | 14.92-29.98 |
|  | Obese | 168 | 101/67 | 38.30 (8.56) | 33.24 (3.70) | 30.00-50.86 |
| FHS Exam 2 | Nonobese | 1108 | 520/588 | 44.59 (9.26) | 24.34 (2.93) | 16.18-29.99 |
|  | Obese | 159 | 88/71 | 45.98 (8.50) | 33.57 (3.80) | 30.04-52.49 |
| FHS Exam 3 | Nonobese | 1098 | 513/585 | 49.14 (9.40) | 24.71 (2.90) | 15.58-29.98 |
|  | Obese | 212 | 112/100 | 49.75 (8.58) | 33.88 (4.17) | 30.01-53.95 |
| FHS Exam 4 | Nonobese | 1133 | 515/618 | 52.37 (9.41) | 25.01 (2.84) | 17.33-29.99 |
|  | Obese | 298 | 166/132 | 53.06 (8.67) | 33.76 (3.94) | 30.02-66.40 |
| FHS Exam 5 | Nonobese | 1081 | 488/593 | 55.97 (9.40) | 25.26 (2.77) | 17.11-29.88 |
|  | Obese | 350 | 192/158 | 56.61 (8.77) | 33.91 (3.86) | 30.01-51.30 |
| FHS Exam 6 | Nonobese | 1020 | 459/561 | 60.14 (9.34) | 25.36 (2.73) | 17.16-29.18 |
|  | Obese | 409 | 224/185 | 59.72 (8.63) | 33.96 (3.87) | 30.00-52.85 |
| Iceland | Nonobese | 3369 | 1490/1879 | 53.43 (16.7) | 24.83 (3.09) | 13.74-29.97 |
|  | Obese | 1818 | 898/920 | 50.61 (16.28) | 35.99 (5.35) | 30.00-72.23 |
| KORA S3 | Nonobese | 3233 | 1643 / 1590 | 47.29 (13.82) | 25.19 (2.78) | 16.44-29.99 |
|  | Obese | 851 | 396 / 455 | 54.04 (12.21) | 33.45 (3.38) | 30.00-56.93 |
| Maywood | Nonobese | 561 | 335/226 | 42.53 (9.56) | 23.77(3.12) | 12.88-29.96 |
|  | Obese | 332 | 95/237 | 43.70 (12.64) | 35.90 (5.00) | 30.01-68.36 |
| Scandinavia | Nonobese | 793 | 448/345 | 67.23 (11.66) | 25.57 (2.56) | 18.36-29.99 |
|  | Obese | 120 | 50/70 | 63.59 (12.34) | 33.01 (2.97) | 30.04-43.34 |
